# Heritability estimates of the position and number of facial hair whorls in Thoroughbred horses

**DOI:** 10.1186/s13104-019-4386-x

**Published:** 2019-06-18

**Authors:** Tamu Yokomori, Teruaki Tozaki, Hiroshi Mita, Takeshi Miyake, Hironaga Kakoi, Yuki Kobayashi, Kanichi Kusano, Takuya Itou

**Affiliations:** 1 Nihon University Veterinary Research Center, Fujisawa, Kanagawa 252-0880 Japan; 2 Genetic Analysis Department, Laboratory of Racing Chemistry, Utsunomiya, Tochigi 320-0851 Japan; 3 Clinical Veterinary Medicine Division, Equine Research Institute, Japan Racing Association, Shimotsuke, Tochigi 329-0412 Japan; 4 Comparative Agricultural Sciences, Graduate School of Agriculture, Kyoto University, Sakyo, Kyoto, 606-852 Japan; 5 Racehorse Hospital Ritto Training Center, Japan Racing Association, 1028 Misono, Ritto, Shiga 520-3085 Japan

**Keywords:** Hair whorl, Heritability, Horse, Thoroughbred

## Abstract

**Objective:**

According to oral traditions of horse caretakers and trainers, the differences in the position and number of facial hair whorls may be associated with temperamental traits. Elucidating genetic background of facial hair whorls and its relationship to temperamental traits may promote more efficient breeding and maintenance of racehorses. In this study, we estimated heritabilities of the position and number of facial hair whorls in Japanese Thoroughbred horses.

**Results:**

The number of facial hair whorls varied from one to four and heritability estimate in 4024 Thoroughbred horses was low (*h*^2^= 0.160). The positions of facial hair whorls were categorized into high, medium, and low, based on their locations. This trait was estimated to have high heritability (*h*^2^= 0.643) in 3782 Thoroughbred horses. These results indicated that a larger proportion of the variation in the studied population was due to genetic factors for facial hair whorls position. Because a similar result was also observed in another horse breed, Polish Konik horses, high heritability of facial hair whorl position may be characteristic of multiple horse breeds. We expect that these results will stimulate future studies to elucidate the relationship among temperamental traits and facial hair whorls in all horse breeds.

**Electronic supplementary material:**

The online version of this article (10.1186/s13104-019-4386-x) contains supplementary material, which is available to authorized users.

## Introduction

In general, horses have about 2-cm-long hairs on their body, and hair whorls exist on various body parts. The number and position of hair whorls have been used for individual identification of horses because they are quite individual. Almost all horses have one or more hair whorls on their forehead, and these facial hair whorls are classified into high, medium, and low, based on their position.

According to oral traditions among horse caretakers and trainers, facial hair whorls may be related to temperamental traits in horses [1, 2]. Horses with a hair whorl at the low position might be difficult to handle, and horses with multiple facial hair whorls tend to fear novel items and environmental conditions [3]. In several recent studies, the relationships among facial hair whorls and behaviors were evaluated in horses. Rotation of facial hair whorl correlates with the turning response [4]. Right-lateralized horses have more clockwise facial hair whorls, whereas left-lateralized horses have more counter-clockwise ones [5].

Interestingly, heritability (*h*^2^) of facial hair whorl position has been estimated to be 0.753 in primitive Konik horse [6], which is a small and semi-feral horse in Poland. However, there have been no such studies in other breeds.

Thoroughbred is a breed produced by crossing Arabian stallions and native British mares at around seventeenth century, and then it underwent selection for superior racing abilities based on speed and stamina [7]. Therefore, while racing performance trait had been selected in their breeding history, temperament trait was not been selected and was variable in this breed. Several temperament phenotypes may affect not only the race performance but also the use of horses after retirement from racing.

In this study, we aimed to estimate heritabilities of the number and position of facial hair whorls in Japanese Thoroughbred horse populations. Elucidating the genetic background of facial hair whorls may inform horse breeders about temperamental traits, which, in turn, should optimize horse utility. We also hypothesized that positions of facial hair whorls are heritable in multiple horse breeds.

## Main text

### Materials and methods

#### Thoroughbred horses

In this study, characteristics of 4845 Thoroughbred horses in Japan were analyzed. These horses were born in 2014 and registered with Japan Racehorse Association (JRA) as racehorses.

Multiple horses were excluded from the original population (4845 horses) for estimating heritability, because of maximum inputs in the GSTM program. Horses in small paternal half-sib families (under 20 or 10 horses at the position and number of facial hair whorls, respectively), and horses with the same phenotypes in their paternal half-sib families were excluded because of less informative.

#### Phenotypic information

**Fig. 1:**
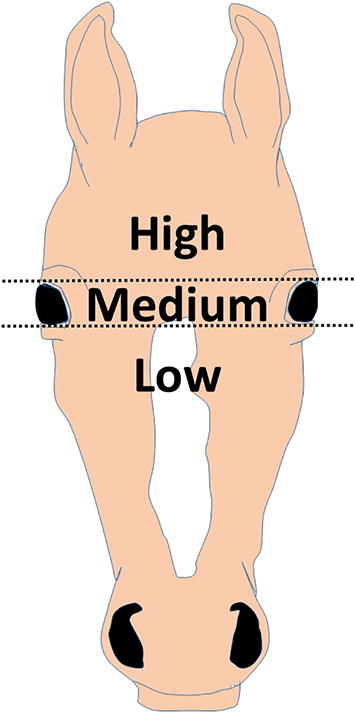
Position of hair whorls on horse forehead. The positions of facial hair whorls were categorized as high, medium, and low. Medium is located on between the eye lines, high and low are located above and below the eye line, respectively

Phenotypic information, namely the number and position of facial hair whorls, sex, and ancestors for three generations (13,057 horses), was collected from the Japan Racing Information System (JARIS), which is a database for JRA racehorses. As shown in Fig. 1, positions of facial hair whorls were classified based on Grandin et al. [8]. Phenotype frequency was manually counted and analyzed.

#### Heritability estimation

The traits (i.e., the number or position of whorls) were analyzed using non-linear categorical (threshold) trait models in Gibbs Sampling Threshold Model (GSTM) program [9]. To estimate heritability, the pedigree information of the Thoroughbred horses was traced back to three generations (maximum inputs in the GSTM program). The following model was used to perform a non-linear categorical trait analysis:

$$ L_{ijk} = SEX_{i} + u_{k} + e_{ijk} $$

where *L*_*ijk*_ is the normally distributed liability assumed with threshold *t* behind the categorical phenotypes *Y*_*ijk*_ of the categorized traits. The category values of *Y*_*ijk*_ begin with 1. For example, in the case of binary phenotypes, the relationship between *L*_*ijk*_, *Y*_*ijk*_, and *t* is as follows: *Y*_*ijk*_ = 1 (*L*_*ijk*_ ≤ *t*) and *Y*_*ijk*_ = 2 (*t* < *L*_*ijk*_). *SEX*_*i*_ is the effect of the *i*th sex, *u*_*k*_ is the breeding value of the *k*th individual [i.e., it follows normal distribution [*N*(**0**, **A**σ^2^_*u*_)], and *e*_*ijk*_ is the residual effect [*N*(**0**, **I**σ^2^_*e*_)]. In these two distributions, **A** is the numerator relationship matrix, σ^2^_*u*_ is the additive genetic variance, **I** is the identity matrix, and σ^2^_*e*_ is the residual variance.

Heritability (*h*^2^) is then defined as σ^2^_*u*_/(σ^2^_*u*_ + σ^2^_*e*_). The total number of Gibbs samplings with GSTM program was 1,050,000, with a burn-in period of 50,000, which was sufficiently large for convergence of the parameters estimated in the present study.

### Results

#### Phenotype of facial hair whorls

As shown in Table 1, Thoroughbred racehorses had one to four whorls. The majority of them had one (81.07%) or two (18.65%) whorls. Accordingly, whorl positions were analyzed depending on whether the horse had one (81.07%) and multiple (18.93%) whorls. Furthermore, 55.77% horses had one whorl at the high position, 22.93%—at the medium position, and 2.37%—at the low position.

**Table 1:** Frequency of hair whorl phenotypes in Thoroughbred horses

Number of hair whorls	Counts	Percentage
1	3928	81.07
2	904	18.69
3	12	0.25
4	1	0.02

Position of hair whorl	Counts	Percentage
High	2702	55.77
Medium	1111	22.93
Low	115	2.37
Multiple	917	18.93

#### Heritability estimates

By eliminating horses in small paternal half-sib families and horses with the same phenotypes as in their paternal half-sib families, 4024 horses that had one (3280 horses) or two (744 horses) whorls and their 10,765 ancestors were used for estimating heritability of facial whorl number. We calculated heritability estimate of whorl number to be 0.160 (Table 2).

**Table 2:** Heritability estimates of hair whorl in Thoroughbred horses

	Mode	Mean ± SD	95% CI
Number	0.160	0.192 ± 0.061	0.093–0.332
Position	0.643	0.660 ± 0.097	0.478–0.852

By eliminating horses in small paternal half-sib families and horses with the same phenotypes as in their paternal half-sib families, 3782 horses with one whorl in the high (2556 horses), medium (1111 horses), and low (115 horses) position, and their 10,592 ancestors were used for estimating heritability of facial whorl position. According to our calculations, heritability estimate of whorl position was 0.643 (Table 2).

The posterior probabilities of the heritability of the whorl number and position showed that those heritability estimates clearly differed from zero and generally distributed normally (Additional file 1: Figure S1), indicating that these are heritable traits.

### Discussion

In this study, high heritability (*h*^2^= 0.643) of facial hair whorl position was demonstrated in Thoroughbred horses. A similar study in the Konik horse population also reported high heritability of this trait (*h*^2^= 0.753) [6]. Because high heritability estimates were obtained independently in different breeds, it can be concluded that a large proportion of the variation in facial hair whorl positions in horses is due to genetic factors.

In contrast, heritability estimate of facial hair whorl number was low (*h*^2^= 0.160), although 95% CI of the heritability did not include “zero”, indicating that variation of facial hair whorl number is affected largely by environmental but also by genetic factors.

High heritability of optimal race distance has been reported in a Thoroughbred population (*h*^2^= 0.51) [10] and *myostatin* (*MSTN*) gene was identified as a single causative gene of this effect by a genome-wide association study (GWAS) using single nucleotide polymorphisms [11]. Moderate heritability of racing performance (win vs. non-win) was estimated in a Japanese Thoroughbred population (*h*^2^= 0.34) [12], and a genomic region spanning *MSTN* was also identified by GWAS by using microsatellites [13]. Hence, it is expected that causative genes underlying facial hair whorl position may be identified in future by GWAS. While heritability of hair whorl number was low, GWAS may also clarify causative genes because the trait may be qualitative (i.e., mainly controlled by a major gene).

Various methods of horse character evaluation have ever been suggested but these may be biased depending on the evaluator [14]. In addition, it is difficult to distinguish between inborn and potentially acquired temperament [15]. Therefore, isolating candidate genes may lead to the development of a genetic test for temperament because of the relatively high heritability of facial hair whorl position. It may also optimize life after racehorse retirement and improve horse welfare.

### Conclusion

In conclusion, our results suggested that genetic factors contribute more to the variability of facial hair whorl position rather than to that of whorl number. These findings could be useful for designing a rational plan of racehorse management. We also expect that these results will stimulate future studies to elucidate the relationship among temperamental traits and facial hair whorls in all horse breeds.

## Limitations

It should be noted that heritability values were estimated using sample populations, in which horses with the same phenotypes as those in their paternal half-sib family and horses in small paternal half-sib families were eliminated.

## Additional file

**Additional file 1: Figure S1.** Posterior probabilities (distribution) of heritability estimates for the number (a) and position (b) of hair whorls. Horizontal axis: heritability values; vertical axis: posterior probability with corresponding heritability value.

## Data Availability

All the data supporting the findings are contained within the manuscript.

## Abbreviations

GSTM: Gibbs Sampling Threshold Model
GWAS: genome-wide association study
JARIS: Japan Racing Information System
JRA: Japan Racehorse Association
*MSTN*: *myostatin*
