# Supplementary material for: Heritability estimates of the position and number of facial hair whorls in Thoroughbred horses
Source: BMC Res Notes. 2019 Jun 18;12:346. doi: 10.1186/s13104-019-4386-x (PMC6582570; doi:10.1186/s13104-019-4386-x)
Supplement: Supplementary file 1 — Additional file 1: Figure S1. Posterior probabilities (distribution) of heritability estimates for the number (a) and position (b) of hair whorls. Horizontal axis: heritability values; vertical axis: posterior probability with corresponding heritability value. [file 13104_2019_4386_MOESM1_ESM.pdf]

a) b)

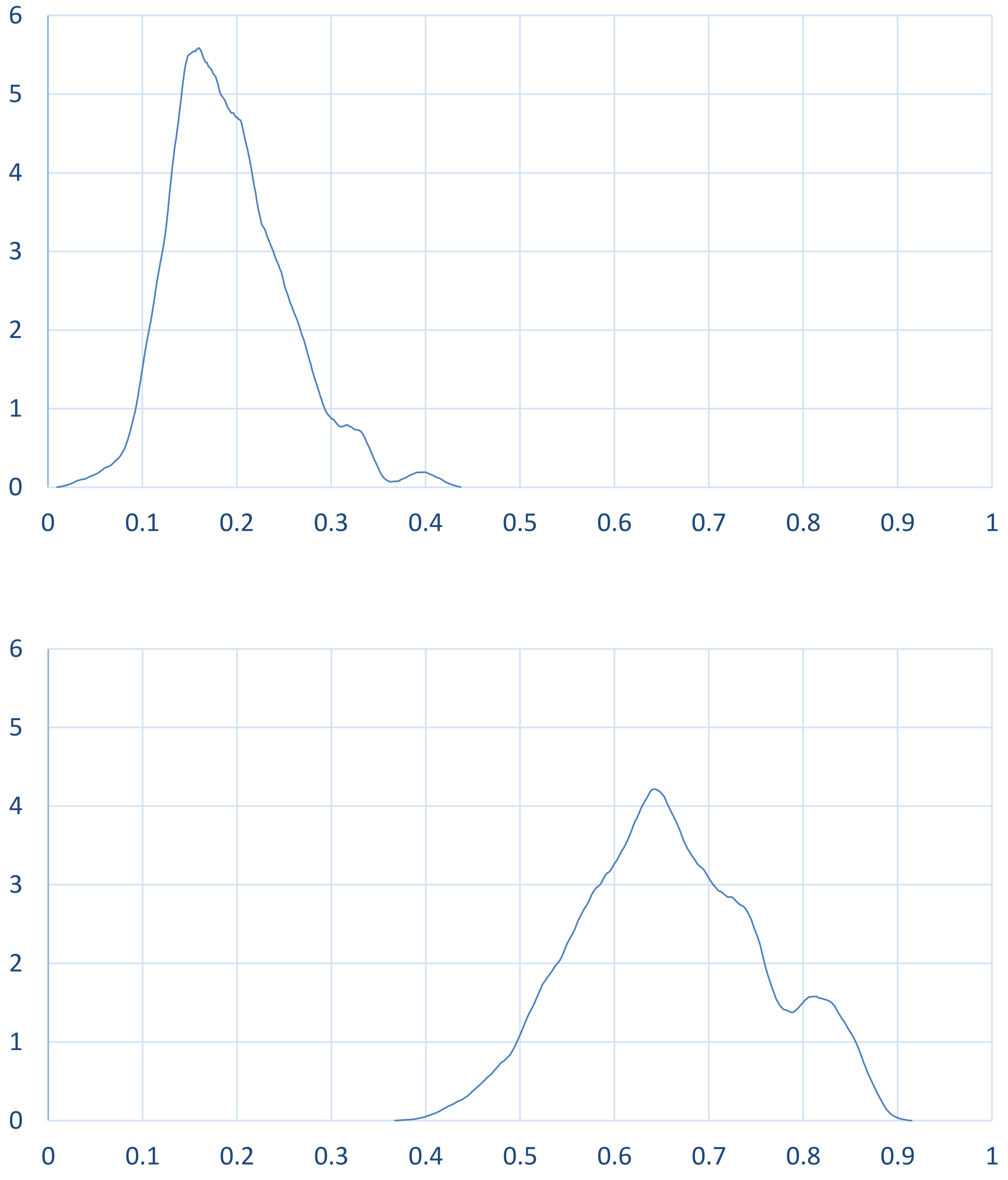

Figure S1. Posterior probabilities (distribution) of heritability estimates for number (a) and position (b) of hair whorls.

Horizontal axis: heritability values; vertical axis: posterior probability with corresponding heritability value.
